# Medical 3D Printing Using Material Jetting: Technology Overview, Medical Applications, and Challenges

**DOI:** 10.3390/bioengineering12030249

**Published:** 2025-02-28

**Authors:** Shivum Chokshi, Raghav Gangatirkar, Anish Kandi, Maria DeLeonibus, Mohamed Kamel, Seetharam Chadalavada, Rajul Gupta, Harshitha Munigala, Karthik Tappa, Shayne Kondor, Michael B. Burch, Prashanth Ravi

**Affiliations:** 1 Department of Radiology, College of Medicine, University of Cincinnati, Cincinnati, OH 45221, USA; chokshsu@ucmail.uc.edu (S.C.); raghav.gangatirkar@gmail.com (R.G.); reddyanish17@gmail.com (A.K.); chadalsc@ucmail.uc.edu (S.C.); ironmanta@icloud.com (S.K.); burchmb@ucmail.uc.edu (M.B.B.); 2 Department of General Surgery, Division of Oral and Maxillofacial Surgery, College of Medicine, University of Cincinnati, Cincinnati, OH 45221, USA; madeleonibus@gmail.com; 3 Department of Urology, College of Medicine, University of Cincinnati, Cincinnati, OH 45221, USA; kamelme@ucmail.uc.edu; 4 Department of Orthopedics, College of Medicine, University of Cincinnati, Cincinnati, OH 45221, USA; gupta2rl@ucmail.uc.edu; 5 Department of Biomedical Engineering, College of Engineering and Applied Science, University of Cincinnati, Cincinnati, OH 45221, USA; munigaha@mail.uc.edu; 6 Department of Breast Imaging, Division of Diagnostic Imaging, The University of Texas MD Anderson Cancer Center, Houston, TX 77030, USA; kktappa@mdanderson.org

**Keywords:** material jetting, medical 3D printing, point-of-care additive manufacturing, systematic review, anatomic models, medical devices

## Abstract

Material Jetting (MJT) 3D printing (3DP) is a specific technology that deposits photocurable droplets of material and colored inks to fabricate objects layer-by-layer. The high resolution and full color capability render MJT 3DP an ideal technology for 3DP in medicine as evidenced by the 3DP literature. The technology has been adopted globally across the Americas, Europe, Asia, and Australia. While MJT 3D printers can be expensive, their ability to fabricate highly accurate and multi-color parts provides a lucrative opportunity in the creation of advanced prototypes and medical models. The literature on MJT 3DP has expanded greatly as of late, in part aided by the lowering costs of the technology, and this report is the first review to document the applications of MJT in medicine. Additionally, this report portrays the technological information behind MJT 3DP, cases involving fabricated MJT 3DP models from the University of Cincinnati 3DP lab, as well as the challenges of MJT in a clinical setting, including cost, expertise in managing the machines, and scalability issues. It is expected that MJT 3DP, as imaging and segmentation technologies undergo future improvement, will be best poised with representing the voxel-level-variations captured by radiologic-image-sets due to its capacity for voxel-level-control.

## 1. Introduction

Medical three-dimensional printing (3DP) has been increasingly examined in the literature for its utility in pre-operative planning, surgical simulation, surgical guidance, surgical training, implants, prosthetics, patient education, and student education [1]. Furthermore, as the costs associated with 3DP decrease, hospitals have begun to establish point-of-care 3DP centers which may be subject to FDA regulation in the near future [2]. The ability to fabricate a wide variety of designs makes 3DP ideally suited for healthcare due to the organic geometries involved as well as the need to personalize models on a patient-specific level. Medical 3DP is commonly carried out through a seven-step method that begins with the acquisition of radiologic images [3]. Next, a process known as segmentation is utilized in which patient medical-image voxel data are clustered by anatomical regions of interest and may be converted into a Computer-Aided Design (CAD) object. Third, CAD-based processing is carried out where the CAD model is smoothed, fixed, and prepared. Fourth, the model is then placed in print preparation software as a Standard Tessellation Language (STL) file for printing on a specific 3D printer. Fifth, the 3D model is converted to instructions for printing via a process referred to as slicing. Sixth, the fabricated model undergoes post-processing where the support scaffolding and any raw material attached to the model is removed. Seventh, the model is then inspected for its quality in which a decision is made whether the model is to be re-printed or utilized in its current form for the target application.

Over nearly four decades, 3DP has proliferated into numerous techniques. Three-dimensional printing was first developed in the 1980s by Hideo Kodama in which he developed the first precursors of stereolithography referred to today as vat photopolymerization (VP) [4]. VP technology utilizes ultraviolet (UV) light that cures a photosensitive resin into a solid object. Charles Hull filed the famous stereolithography apparatus (SLA) patent in 1986 that is generally regarded to mark the founding of 3DP [5]. Following Kodama’s work, selective laser sintering (SLS) 3DP, where fine polymer powder particles are fused through sintering, was developed by Carl Deckard. Material extrusion (MEX), where molten plastic filaments and/or pellets are deposited through heated extruders, was developed by Scott Crump [6]. Following the 1980s, the 1990s oversaw the development of many other 3DP technologies such as Powder Bed Fusion (PBF), Binder Jetting (BJT), Sheet Lamination (SL), Material Jetting (MJT), and Directed Energy Deposition (DED).

Among these technologies, MJT presents a great advantage in its potential ability to not only print 3D models with a high level of accuracy, but also print multi-part objects with a controllable degree of transparency, elasticity, and color previously unavailable in 3DP [7]. MJT was first developed by Solidscape (then called Sander’s Prototype) in the 1990s, in which wax droplets were deposited using a mechanically positioned inkjet printer head [8]. In 1996, 3D Systems developed the Actua 2100, a comparable MJT 3D printer that utilized similar heated waxy thermoplastic material. The technology of MJT 3DP further developed in the 2000s with the 3D printer Quadra by 3D Systems in 2003 as well as the Polyjet^ä^ line of 3D printers developed by Objet in 1999. Furthermore, in 2012, Stratasys merged with Objet, continuing their line of 3D printers in addition with starting a new line of Medi-Jet^ä^ and Denta-Jet^ä^ MJT 3D printers in 2020. It must be noted that while companies have named their respective technologies differently, these all refer to MJT 3DP technology. MJT 3D printers utilize photopolymers in which the printer head deposits materials that are then polymerized with UV radiation [9]. This relatively new technology was noted to produce objects with a 28 μm layer height. In some specialized printers, such as the Stratasys J850, the minimum layer height is reported at 14 μm. Recently, 3D printers that utilize MJT have emerged that can print in metal or ceramic materials [10].

While many literature surveys exist that investigate medical 3DP, no reviews focusing on MJT medical 3DP have been written thus far to our knowledge. In 2020, a review was written of literature involving medical 3DP used in the pre-operative planning of surgical procedures [11]. A 2023 literature review investigated various 3D printing technologies within personalized medicine, biopharmaceuticals, and nanomedicines [12]. A similar research paper was written two years prior in 2021 that investigated MEX, SLS, and VP 3DP of polymers [13]. Two further literature reviews were written in 2021 in which the first investigated various 3DP technologies in their ability to construct models for craniofacial applications in rehabilitation, reconstruction, and regeneration, and the second investigated 3DP medical devices used to treat patients directly [14,15]. Two literature reviews written in 2023 investigated applications of 3DP in the field of biomedical engineering as well as in the preprocedural planning for left atrial appendage occlusion [16,17]. Many additional reviews have been written that investigated previous 3DP applications within specific fields of medicine that apply to orthopedics [18], cranio-maxillo facial surgery [19], neurosurgery [20], urology [21], ophthalmology [22], as well as liver surgery [23], among other fields. A recent review of VP medical 3DP investigated various clinical applications, accuracy studies, and challenges of the technology [2].

This review article provides a comprehensive overview of numerous medical applications in which MJT 3DP has previously been used in the literature as well as in our own lab housed at the University of Cincinnati and provides an overview of MJT 3DP technology. This review does not adhere to the conventional structure of a systematic review, but rather illustrates an extensive assessment of medical MJT 3DP coming from expert practitioners.

## 2. Material Jetting (MJT) 3D Printing Technology Overview

MJT technology utilizes air-excluding reservoirs to store photo polymeric materials, which are then deposited as droplets forming slender layers on the build platform. Colored inks can also be included as photo polymeric material is deposited to provide full color print capability. To facilitate the curing of this deposited material, UV light is then projected on the build platform. Upon curing each layer, the build platform is incrementally lowered by a predetermined layer thickness, and fresh liquid material is jetted onto the preceding layer. Through this iterative process of curing successive layers, a complete part can be fabricated. However, in certain structures with overhangs, support material in a wax-like form is necessary to maintain the integrity of the printed object. The elimination of these support structures from the final part can then be carried out with many different techniques such as model immersion in a sodium hydroxide solution bath with sonication, application of heat, or employment of a high-pressure water jet. However, the surfaces where support material contacts the model will have a “matte” finish and this will require polishing and a coat of lacquer or other similar liquid in transparent models to achieve optimal optical transparency. Removal of support structures from the model using high pressured water can damage intricate features in the model. However, the latest generation of support material can be dissolved in water and/or lye.

Because MJT works by jetting tiny droplets of photocurable material from an array of nozzles, it is possible to mix and match different materials to obtain a range of different mechanical and optical properties. Since radiologic images contain voxel data with density information, MJT may be best suited to fully translate that digital data into a physical model owing to its ability to tightly control the process at a voxel level [24]. Additionally, with the development of materials that better mimic tissue mechanical properties, it may be possible to fabricate anatomic models that more closely match the visual as well as tactile characteristics of native tissue to provide the physician with a highly realistic simulation prior to the surgery.

**Figure 1:**
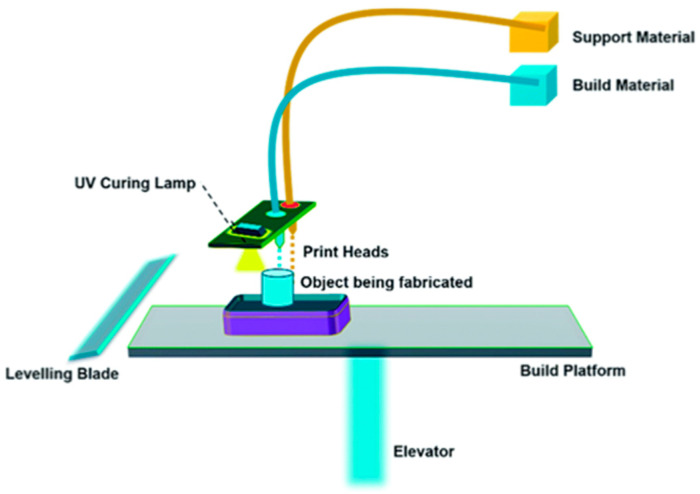
Schematic of a typical MJT 3D printer. Droplets of photopolymers and supporting material are jetted onto the build platform in a layer-by-layer fashion. The print heads translate on a gantry along two axes whereas the build platform typically translates along a single vertical axis. The UV lamp hardens material after ejection. Colored inks provide full color printing capability (From Ref. [25], used under Creative Commons CC-BY 4.0 license).

**Figure 2:**
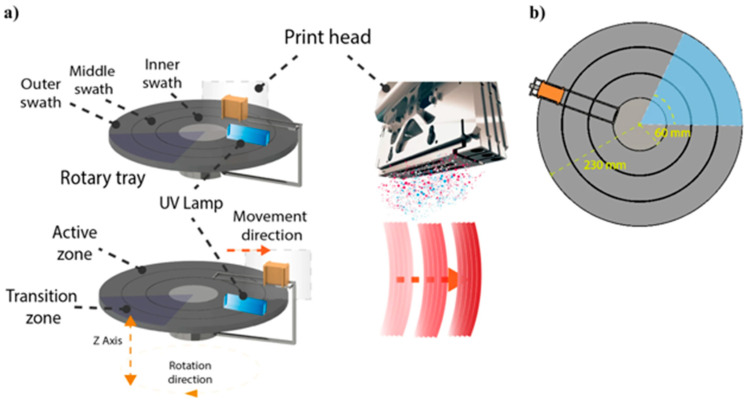
(**a**) Schematic of a more recent MJT 3D printer with rotating build tray. The printheads translate radially and the build platform rotates continuously. The build platform also translates vertically allowing for the layer-by-layer fabrication. (**b**) Because the tangential velocity increases with distance from the center of the build platform, models placed in the inner swath print the fastest (From Ref. [28], used under Creative Commons CC-BY 4.0 license).

An illustrative diagram is portrayed above that provides a general overview of how most MJT 3DP are structured (Figure 1) [25]. However, this representation can differ depending on manufacturer or 3DP model. In fact, MJT is referenced as PolyJet^ä^ in Stratasys 3D printers and as MultiJet^ä^ in printers from 3D Systems. However, per ASTM F42 standard terminology, MJT is the appropriate term to refer to this umbrella of technologies with slight variations between vendors and printer models [26]. One example of such a difference in MJT 3D printers can be seen in the Stratasys J5, which utilizes a rotating build tray such that the heads that jet material move only in the radial direction and not in the two dimensions as is the case with the typical MJT gantry-based printers [27]. Relatively smaller models that can be accommodated in the innermost swath (Figure 2) will print faster compared to larger models that span across multiple swaths due to the higher tangential velocity [28]. This necessitates multiple passes from the print head (multiple rotations) to successfully dispense the defined volume of material as well as to fully cure it in place with the UV lamp.

In terms of its throughput, MJT technology is on par with other technologies and maybe better when factoring in the accuracy and surface finish of the printed part. However, 3D printing technologies are, in general, slow because of the low volumetric material feed rate inherent in these processes. The cost of MJT 3D printing can represent a substantial capital investment to procure the printer and materials, but the printer prices are reducing now with the miniaturization of the technology and its export to desktop 3D printing as demand continues to grow.

## 3. Literature Review

MJT 3DP has been used in a variety of clinical applications owing to its ability to fabricate high quality and accurate models in full color detail (Table 1). The clinical applications are categorized by organ system in this section. Our comprehensive literature search was performed on the PubMed National Library of Medicine with the following search terms: (3D Printing and Medicine and Material Jetting) or (Additive Manufacturing and Medicine and Material Jetting) or (Rapid Prototyping and Medicine and Material Jetting) or (3D Printing and Medicine and PolyJet™) or (Additive Manufacturing and Medicine and PolyJet™) or (Rapid Prototyping and Medicine and PolyJet™). Of the 99 articles identified in this search, 62 articles were selected based on abstract screening for relevance to clinical applications.

### 3.1. Liver

**Figure 3:**
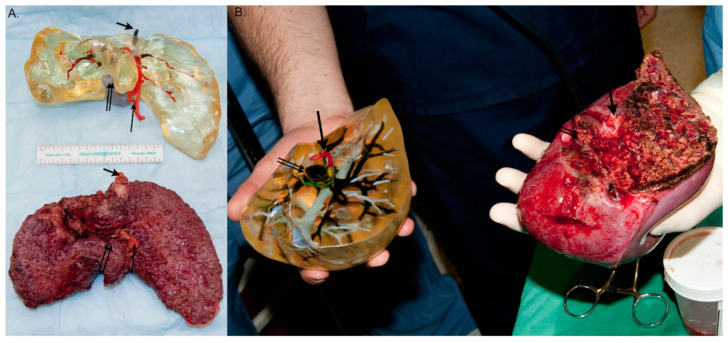
(**A**) An explanted liver compared with its represented 3D model. Within the image, the short, wider arrows point to the hepatic vein, the long, thin arrows point to the hepatic artery, and the long, thin, double arrows point to the portal vein. (**B**) A comparison between the right lobe and actual right lobe of the donor liver. Within the image, the double arrows point at the portal vein, and the single arrows point to the hepatic artery (From Ref. [29], used under Creative Commons CC-BY 4.0 license).

The first noted liver application of MJT 3DP occurred in 2013 in which a MJT 3D printer was applied in the creation of full color models used in the pre-operative planning of three living donor liver transplantation (LDLT) cases (Figure 3) [29]. Researchers involved in the study found the models to be highly accurate as well as superior to 2D liver visualization with medical imaging; however, they also noted that production of a single model involved 25–30 h of labor in addition to the high material cost.

Following 2013, the utility of MJT 3DP in the pre-operative planning as well as patient education of liver surgery was further investigated. One comparable study in 2015 noted the benefit of utilizing a MJT 3DP transparent liver model (to visualize the hepatic and portal veins as well as the tumor) in pre-operatively forming a treatment plan for a pediatric case of hepatoblastoma [30]. An additional study in 2016 noted the value of MJT multi-color printing in the formation of models to pre-operatively plan LDLT in infants [31]. Furthermore, liver MJT 3DP was defined as accurate and highly detailed through a 2017 feasibility study in which the investigated model included a portal vein with a tumor, a hepatic vein, a hepatic artery, and a biliary tree with a gallbladder [32].

### 3.2. Prostate, Kidney, and Pelvis

Three-dimensional printed models fabricated with MJT technology have been reported to be useful in surgical planning of robotic-assisted radical prostatectomy [33]. In the 18 patient study, favorable feedback for the prostate models were provided by patients as well as surgeons. A similar study focusing on patient education regarding kidney and tumor anatomy additionally found favorable results through a pre- and post-test associated with use of the kidney MJT 3DP model [34]. On the other hand, a research article published in 2018 noted that use of an MJT 3DP kidney model was useful, paired with other imaging modalities, intra-operatively for a complex kidney stone case [35]. Additionally, an MJT 3DP model utilized in a case report of a patient with endometriosis was noted to have been accurate in terms of anatomy as well as useful in surgical planning [36]. The utility of MJT 3DP has also been established in robotic-assisted partial nephrectomy from a study in 2018 [37]. The authors in the study demonstrated its utility through portraying a successful patient case in which the model was used to assist in surgery preoperatively and intraoperatively. While MJT 3DP has substantiated itself positively among the previous medical applications in the prostate, kidney, and pelvis, a recent paper revealed that attending urologists (n = 180) found MJT 3DP to be poor compared with MEX in determining prostate anatomy and tumor location [38].

### 3.3. Oral and Cranio-Maxillofacial

The anatomic variation associated with conditions in oral and cranio-maxillofacial pathologies make them subject to potential applications with MJT 3DP. Researchers have found MJT 3DP models to be accurate and have utility in the pre-operative planning of treating complex intracranial aneurysms [39], treating complex deformities of the skull base and craniovertebral junction [40], planning pediatric mastoid surgery [41], treating mandibular prognathism [42], and planning skull-base and tumor surgery [43].

### 3.4. Ophthalmology

The medical applications of MJT 3DP in ophthalmology are extensive. Kozakiewicz et al. [44] presented a method to overcome the difficulty associated with fitting and aligning implants used to treat orbital floor implants. The authors utilized MJT 3DP to create patient specific models that titanium meshes would be pre-bent on for six patient cases. The authors reported that this method was financially viable. A comparable study fabricated MJT 3D models to assist in orbital defect reconstruction in which authors reported that the models had clinical utility [45]. Xie et al. in 2014 investigated the utility of MJT 3DP in the creation of an eye model for fundus viewing [46]. It was reported that the eye model could properly simulate the optical performance of a human eye. A similar research article by Adams et al. detailed the feasibility fabrication process for a dissection eye model used in medical student training created with MJT [47]. The authors reported that the model properly portrayed anatomic features. An additional article in 2016 detailed the fabrication with MJT and successful fitting of an ocular prosthesis in a 68-year-old male patient with acquired anopthalmos [48]. A similar ocular prosthesis was printed in 2017 with MJT 3DP in which the patient found the prosthetic to be more comfortable [49]. On the other hand, MJT 3DP was used to create eyelid crutches to treat blepharoptosis at a low cost with success [50].

### 3.5. Phantoms

**Figure 4:**
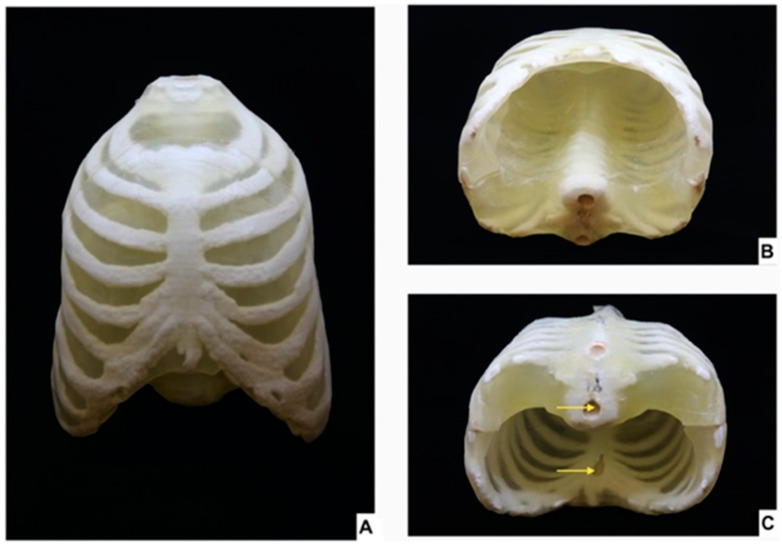
A 3D printed phantom of the thorax. (**A**) Ventral view. (**B**) Caudal view supine position. (**C**) Caudal view prone position in which the yellow arrows represent the inlets in which radiopaque material is placed (From Ref. [52], used under Creative Commons CC-BY 4.0 license).

MJT 3DP has also been applied in the fabrication of 3D printed imaging phantoms. Previous literature includes studies that detail the creation of molecular imaging phantoms [51], an MJT-formed anthropomorphic thorax phantom [52], validate the accuracy of a thyroid cancer phantom created with MJT as opposed to with MEX or color jet printing [53], fabricate soft tissue phantoms to test radiation attenuation in CBCT with MJT 3DP (Figure 4) [54], form cardiovascular phantoms [55], and create a phantom of a glenohumeral joint [56]. Furthermore, additional studies have investigated the best filler compounds as well as imaging properties for MJT 3D printed phantoms to achieve radiopacity [57,58].

### 3.6. Simulators

Surgical simulators require a certain degree of accuracy, realism, as well as functionality to be applied in the training of physicians. While conventional simulators have been developed that hit each of these marks are expensive, MJT 3DP enables the development of patient-specific simulators which may capture further nuance. Many studies were noted in the medical MJT literature which investigated the utility of such simulators in diverse medical indications.

**Figure 5:**
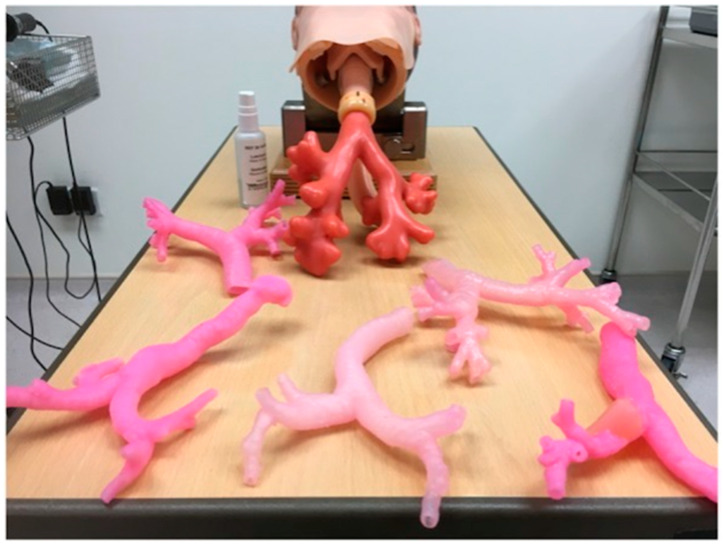
Represented is a standard airway trainer with a detachable airway model in addition with a plethora of 3DP airway structures of cases with diverse clinical indications (From Ref. [59], used under Creative Commons CC-BY 4.0 license).

Bronchoscopic simulators were developed through MJT 3DP of the airways from imaging data of a patient with healthy airways, a patient with a tumor present in one, and a patient with a goiter causing external tracheal compression (Figure 5) [59]. The models were manufactured with flexibility as well as rigidity utilizing MJT ability to print with many materials. Physicians who tested each of the simulators noted that the ones formed with 3DP were suitable for training and have learning value. Hong et al. investigated the utility of 3DP in the creation of pediatric video assisted thoracoscopic surgery (VATS) simulators [60]. The authors printed models with MJT 3DP that included the esophagus, lung, trachea, chest wall, bone, skin, and muscle, as well as seven holes for placement of viewing ports, from medical images of a three-year-old patient. The physicians involved in the study commented that the simulators were realistic and very useful in training as well as pre-operative planning. Simulators also have been utilized in the field of neurosurgery to simulate burr hole procedures. Researchers investigated the utility of eight different 3DP materials across four different 3DP technologies, which included MEX, VP, MJT, SLS, in the fabrication of burr hole simulators. Each of the 3DP technologies would be ranked utilizing criteria of quality of mechanical drilling, visual appearance, skull exterior, and skull interior based on simulation on models by five neurosurgeons on each type of model. The authors found that 3DP technology utilizing MEX as well as VP had higher utility in skull replication as compared with MJT and SLS [61]. MJT 3DP was also applied in rhinoplasty surgical simulators in which a three component device was fabricated that generated five learning point areas [62]. After testing of the simulators, the authors remarked that the simulators were feasible, accurate, as well as low-cost.

MJT 3DP simulators have also been developed in the field of dental and cranio-maxillofacial surgery. Two research articles detailed the fabrication of endoscopic sinus surgery simulators [63,64]. The authors in each of the studies commented on how the MJT technology produced highly accurate and useful in surgical training. Narayanan et al. examined the feasibility in fabricating a simulator for endoscopic skull-base surgery [65]. Examiners within the study commented on the effectiveness of the MJT printed models in training under the supervision of experts. Rose et al. commented on how MJT 3DP had been utilized successfully in the fabrication of simulators for temporal bone surgery [66]. On the other hand, researchers in 2022 examined various different 3D printer technologies (MJT, MEX, VP, and SLS) in their ability to print dental implant surgical simulators [67]. The researchers concluded that the MJT and VP created simulators provided the best haptic realism.

### 3.7. Miscellaneous

A 2020 study applied MJT 3DP in order to fabricate a tooth to be used in transplantation [68]. The authors reported that the post-operative course of the patient in the study was uneventful. On the other hand, an article in 2019 recorded the feasibility and potential of 3DP models that replicated laryngotracheal stenosis with MJT technology [69]. Retrospective findings from the study led the authors to conclude that the MJT fabricated models were feasible in pre-operative planning as well as in assisting with patient education. Along the same lines with education, MJT 3DP has also been previously applied with success in the development of a heart model utilized in learning anatomy [70]. Bergquist et al. in 2019 utilized MJT 3DP in order to fabricate a model used in mapping chest wall instability to assist in determining operative approach through thoracotomy [71]. Unkovskiy et al. in 2021 utilized MJT 3DP to fabricate a sports mouth guard through a proof of concept case [72]. The authors reported that the mouth guard was clinically acceptable. On the other hand, Parthasarathy et al. discusses the fabrication of MJT 3DP musculoskeletal models of pediatric patients with Ewing’s sarcoma, osteosarcoma, and chondrosarcoma [73]. The authors reported that these custom models were pivotal in preoperative planning and intraoperative execution, yielding a mean disparity of −0.09 mm between the models and actual surgical resection.

### 3.8. University of Cincinnati Radiology 3D Printing Lab Cases

**Figure 6:**
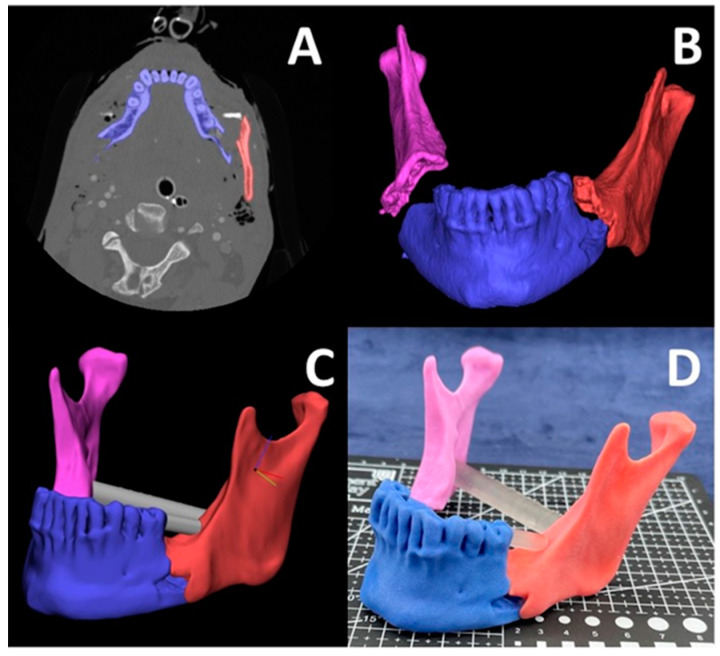
Bilateral open comminuted mandibular angle fractures requiring reduction with plating. (**A**) The axial CT scan with segmented masks of the symphysis (blue) and left angle (red). (**B**) 3D visualization of the segmented masks showing the bilateral mandibular angle fractures including the right mandibular angle (magenta). (**C**) The reduced 3D model in CAD software with pins for structural reinforcement, and (**D**) the final full color 3D printed using MJT technology.

**Figure 7:**
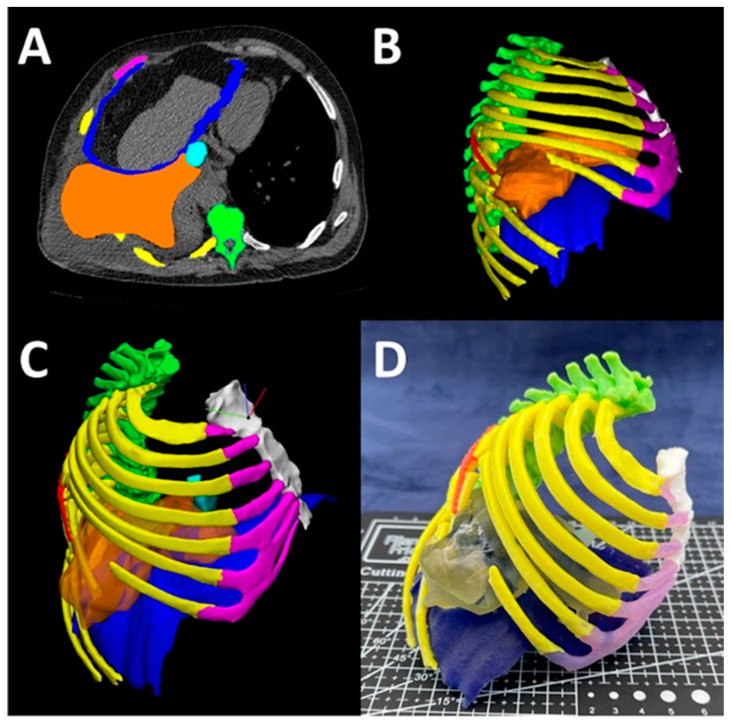
MJT 3DP anatomic model for complex chest wall and diaphragmatic hernia repair planning. The segmented anatomy included the spine (green), ribs (yellow), costal cartilage (magenta), sternum and manubrium (white), the inferior vena cava (cyan), the right hemidiaphragm (blue), the metal plate (red), and the herniated fat (orange). (**A**) The axial CT scan with segmented masks. (**B**) 3D visualization of the segmented masks. (**C**) The digital 3D model in Materialise 3-Matic 18 CAD software, and (**D**) the final full color 3D printed using MJT technology at 33% scale.

**Figure 8:**
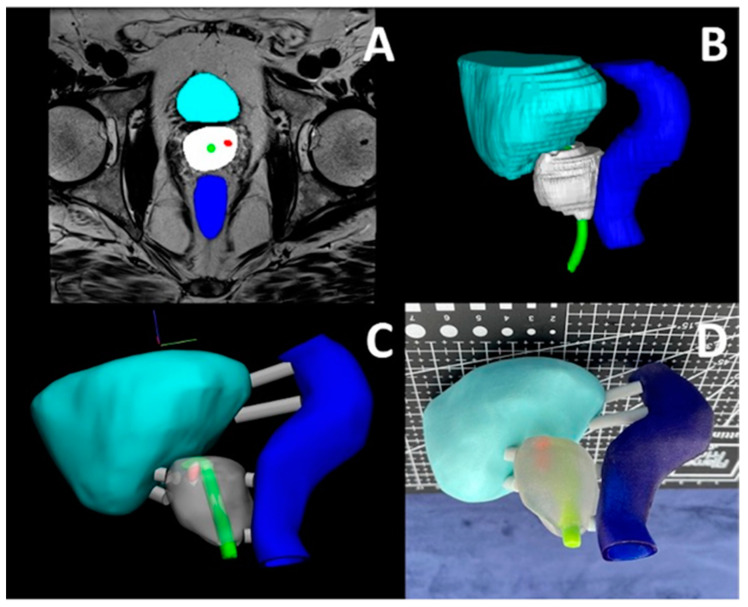
Full color MJT 3DP anatomic model for prostate cancer cryoablation planning. The segmented anatomy included prostate (white), urethra (green), bladder (cyan), rectum (blue) and lesion (red). (**A**) The axial MRI scan with segmented masks. (**B**) 3D visualization of the segmented masks. The MRI slice is visible due to the relatively larger slice thickness. (**C**) The smoothed digital 3D model in CAD software with the urethra and lesion visible through the transparent prostate, and (**D**) the final full color 3D printed using MJT technology.

**Figure 9:**
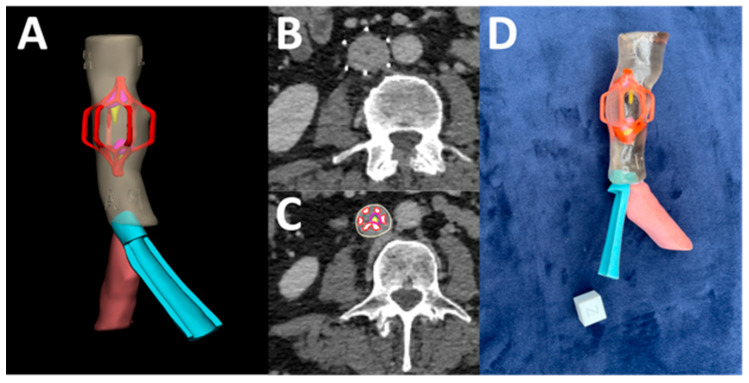
MJT 3DP anatomic model for planning of inferior vena cava filter (IVC) removal. (**A**) 3D model of the segmented masks. (**B**,**C**) Radiological images of the point of interest including segmentation. (**D**) final full color MJT 3DP model placed near calibration cube (1.5 cm × 1.5 cm × 1.5 cm).

**Figure 10:**
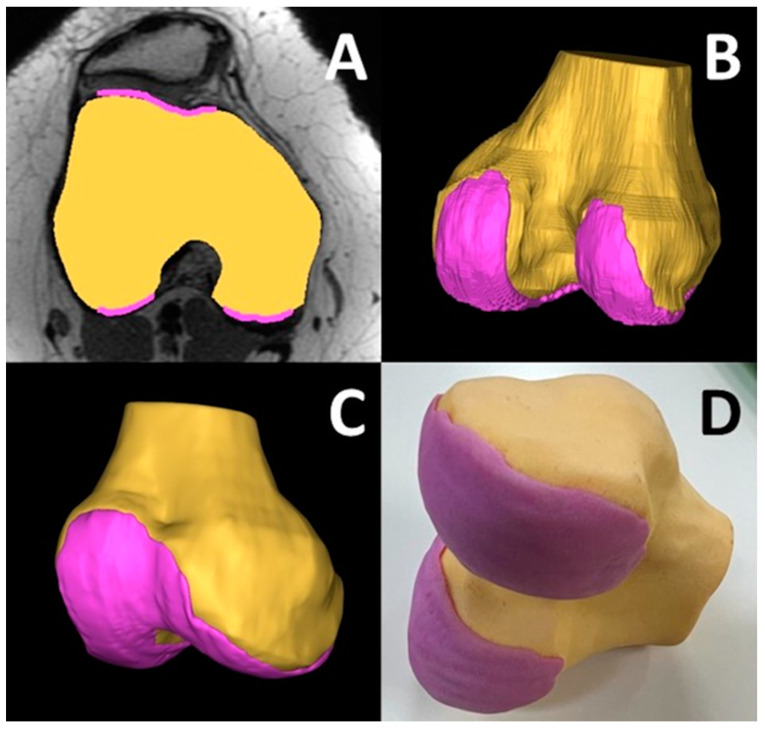
MJT 3D printed distal femur model for three-dimensional visualization of the abnormal anatomy of the trochlear groove for a 20-year old patient. (**A**) Segmented cartilage (magenta) and distal femur (yellow) masks overlaid on axial cube MRI. (**B**) 3D visualization of the segmented masks, (**C**) smoothed surface mesh generated from the masks showing the trochlear groove (**D**) the 3D printed model showing the cartilage and femur. The degree of abnormality often determines the severity of patellar instability. Three-dimensional printed distal femur can potentially outperform CT and MRI for operative planning.

Our 3D printing lab utilizes MJT 3DP using the J5 MediJet 3D printer (Stratasys, Eden Prairie, MN) alongside other 3DP technologies. We have created models for numerous medical applications including mandibular fracture reduction (Figure 6), complex hernia repair (Figure 7) and prostate cancer cryo-ablation (Figure 8), inferior vena cava retrieval (Figure 9), and treatment of patellar instability (Figure 10). The process starts with a medical imageset (CT/Magnetic Resonance Imaging—MRI) and a consult with the ordering physician to understand the medical challenge. This initial consult helps define the requirements of the ordering physician and determines the anatomy to be ultimately segmented to aid in visualization and planning. Once the anatomy is segmented and a surface mesh (STL) is constructed around the segmented volume, a trained radiologist can overlay the contours of the STL onto the source imageset and validate the anatomical accuracy of the digital model. Then, this model is 3D printed and handed off to the ordering physician for pre-procedural planning and/or patient counseling.

### 3.9. Mandibular Fracture Reduction

Recently, a collaboration with our surgeons and our 3DP laboratory was utilized to better aid in surgical planning for a complex facial trauma case. This case was of a patient who presented with injuries sustained from a motorcycle collision where the injuries involved included severely displaced, comminuted fractures of both the right and left mandibular angles. Utilizing Computed Tomography (CT) imaging and 3D rendering alone was quite limiting due to the severity of the fractures. Having the ability to work with the engineers and radiology department at University of Cincinnati to virtually reconstruct and reduce the fractures into appropriate anatomical position allowed the surgical facial team better means of visualizing the needed surgical outcomes. Furthermore, being provided with a full color, three-dimensional model of the reduced mandibular fractures enabled our surgeons to appropriately orient the bony segments intraoperatively and ensured that adequate reduction would be obtained. Having the major fracture segments distinguishable by color variation also provided additional benefits to the case in that our surgical team was able to note intraoperatively whether further dissection was needed to locate and expose any additional bone fragments, and allowed our team to know where the displaced and comminuted fragments were to be placed for adequate anatomical reduction to ensure minimal to no bone defect remained. This model being available prior to surgery allowed for adequate planning of our surgical approach; it provided our team with the confidence and assurance that approaching this case via a transcutaneous approach would provide the most optimal outcome in adequately visualizing, exposing, and reducing the mandibular fractures for the patient. Having this fracture-reduced model also enabled our team to save time in the operating room; we utilized the full color model to visualize how the mandible potentially appeared prior to injury. This allowed our team to determine the appropriate plates and hardware best suited for the case. In doing so, we were able to select pre-bent plates that were already well-adapted to the mandibular model; thus minimal plate bending was required in the operating room. Two key factors in correcting facial trauma are to ensure function is restored and that facial projection is maintained and/or restored. Specifically with mandibular trauma, ensuring occlusion is appropriate, stable, and reproducible post-operatively is vital for the success of the surgery. Additionally, ensuring the maxillofacial buttresses in the vertical and transverse dimensions are maintained or restored is also as equally important because these facial struts provide the framework for the face. Given the complexity of the facial fractures, the displacement and the comminution that were involved, having the ability to study the simulated model prior to surgery provided additional means in ensuring that the goals of the surgery were achieved without compromises to the patient.

### 3.10. Complex Hernia Repair

An additional collaboration with our 3DP lab was fabricating a model treating a complex hernia with chest wall and diaphragmatic reconstruction. In this case, the patient presented with diaphragmatic hernia following failed chest wall hernia repair. To assist in the pre-operative planning and communication of the procedure between two surgeons, an MJT 3DP model was fabricated (Figure 7). Following chest wall reconstruction with mesh, a complete pulmonary decortication was performed due to a thin rind on the right lower, middle, and upper lobe. Furthermore, in diaphragmatic reconstruction, it was noted that fully closing the hernia defect was not possible. The patient had less than 100 mL blood loss and no complications were noted.

### 3.11. Prostate Cancer Cryo-Ablation

With the current evolution of technology in the management of clinically significant prostate cancer veering away from traditional prostatectomy and prostate gland radiation, the role of 3D printing for prostate models is magnified. Newer technological advances in the management of prostate cancer aim to target the affected area in the prostate gland with cancer and spare the apparently normal prostate tissue. This helps to reduce the devastating quality of life-related complications following traditional prostate cancer treatments, notably erectile dysfunction and urinary incontinence. Such newer technologies include ablating the affected prostate tissue using High Intensity Focused Ultrasound (HIFU), Cryosurgery and Irreversible Electroporation (IRE). As with any new technology, the patient usually needs more education and counseling about a newly adopted procedure. The 3D printing of a prostate model, showing and allowing the patient to hold it in his hands and demonstrate the sole location of the cancer and combining it with color coded pathology grade, immensely helps patient education and shared decision making between the patient and physician. It also provides reassurance to the patient regarding the treatment decision. In addition, the ablation of the affected prostate area with cancer is often performed in a cognitive manner. Indeed, the availability of a 3D printed model significantly helps in surgical planning and probe placement to conduct prostate cancer treatment. In addition, in HIFU technology for prostate cancer treatment, the energy is transmitted in zones of the prostate gland with zone overlapping. Identifying which side of the gland is involved (right or left lobe), and which zone; anterior or posterior allows for zone overlapping in the involved area of the prostate gland with intensifying energy to the affected zone—potentially eradicating the affected prostate tissue of cancer. Additionally, identifying the proximity of the prostate cancer-involved area to vital structures, such as the rectum posteriorly, urethra in the middle and external urinary sphincter at the apex of the prostate gland, helps to safeguard these structures during energy administration.

### 3.12. Complex Inferior Vena Cava (IVC) Filter Removal

Furthermore, our 3DP lab collaborated with interventional radiology to perform the removal of an IVC filter. The case involved a 59-year-old female patient with factor V thrombophilia, a pulmonary embolism from deep vein thrombosis, and May-Thurner syndrome, in which complications arising from an indwelling IVC filter and iliac thrombus lead to lower extremity symptoms. In the surgical treatment for this patient, it was noted that the MJT 3DP model (see Figure 9) assisted in preoperative planning for the complex filter removal along with anatomical assessment of an existing stent, giving surgical plans for recanalization of IVC and bilateral veins, a thrombectomy, an angioplasty of the primary right iliac, and a repeat stenting of the left iliac veins.

### 3.13. Patellar Instability Visualization

Patellar instability occurs when the patella (kneecap) moves outside the trochlear groove. In a young patient this can cause pain, swelling, stiffness, difficulty walking on the affected limb, and/or a bucking, catching, or locking sensation in the knee. There could also be a noticeable deformity in the affected knee as well as cracking or popping sounds in the knee when climbing stairs or bending the knee. A MRI is useful in assessing injuries such as tears in the anterior cruciate ligament (ACL) and meniscus or loose bone fragments. Patellar dislocations affect 5.8–29 out of 100,000 females between 11 and 17 years of age. Overall annual incidence has substantially increased in the last 20 years [74]. Patellofemoral joint pathology is complex and difficult to treat. The geometry of the trochlear groove has a major role in patellofemoral instability. Conventional radiography, CT, and MRI are used to define the trochlear anatomy. While useful for planning surgical interventions, each of these modalities has its own limitations. Plain radiography fails to describe the bony surface anatomy of the trochlear groove to account for the rotation of the trans epicondylar axis and posterior condyles. While CT and MRI are more useful, they are still 2-dimensional representations of complex 3-dimensional anatomy and fail to account for the oblique, dysplastic patellar tracking path. Three-dimensional imaging and printing in orthopedic surgery have diverse applications, including preoperative planning, patient-specific instruments, and implants. In the realm of patellofemoral instability, it has the potential for enhanced diagnostic and surgical decision making for complex anatomical issues. Trochlear dysplasia is classified by the globally accepted four-class Dejour system. However, the inter- and intra-observer reliability of the Dejour classification has ranged from poor to modest [75,76]. Consequently, the Dejour classification remains poorly validated. Further, studies have shown that its reliability reduces in cases of severe trochlear dysplasia [77]. It has been concluded that Dejour classification may be misleading if used in clinical and research settings [78]. Three-dimensional printed models may provide a comprehensive visuospatial depiction of complex trochlear anatomy. Recent studies have used CT-based 3D printed anatomic models and have found that it can depict the increased obliquity of the angle that the patella enters into the trochlear groove when it first engages, leading to maltracking and the classical “J” sign [79]. Further, subtle, but important changes in the morphology of the medial trochlear facet have been observed [78]. As part of an ongoing trial to assess the utility of 3D printing in better understanding patellar instability, MJT 3D printing was used to visualize the bone and cartilage in a 20-year old patient (Figure 10) using MRI images for segmentation.

### 3.14. Medical Accuracy Studies

The accuracy of material jetting 3DP in the medical field has been substantiated in the literature (Table 2). Chen et al. investigated the accuracy of various 3DP technologies in the fabrication of surgical templates [80]. Among the technologies, MJT was listed as the most accurate with the lowest Root Mean Square (RMS) value. Kim et al. utilized calipers to determine the accuracy between maxilla and mandible surgical guides fabricated with various technologies [81]. The study found that MJT 3DP had the greatest degree of accuracy. Wang et al. investigated the accuracy differences between various 3DP technologies through a meta-analysis [82]. The article revealed that MJT and SLS 3DP technology fabricated models with the lowest mean difference in terms of accuracy. Further studies found MJT 3DP accuracy to be comparable with other 3DP technologies in surgical as well as dental settings [83,84,85,86,87,88,89,90].

### 3.15. Potential Challenges

As mentioned previously, MJT 3DP has emerged as a transformative tool in healthcare, offering capabilities for creating precise anatomical models and patient-specific surgical guides. However, its integration into clinical practice is not without significant challenges.

### 3.16. High Cost of Equipment and Materials

One of the primary barriers to the widespread adoption of MJT in healthcare is the substantial financial investment required for both the printer and the associated materials used. The initial costs can be prohibitive for many institutions, especially smaller healthcare facilities. Additionally, the expense of biocompatible materials often exceeds that of alternative 3DP technologies, limiting accessibility and scalability.

### 3.17. Material Limitations

While MJT 3DP supports the use of various materials, the selection of biocompatible and biomimicking materials suitable for numerous clinical applications remains limited. The material shore hardness, their shelf-life and long-term performances must be thoroughly understood to ensure their efficacy in clinical scenarios including tissue mimicking modeling and prosthetics.

### 3.18. Technical Expertise

Effective operation and maintenance of MJT 3D printers require a specialized skill set that may not be readily available in all healthcare institutions. This expertise is essential for troubleshooting technical issues, optimizing print settings, and ensuring consistent quality in printed products. The need for trained personnel can pose a challenge, especially in environments where resources are already stretched.

### 3.19. Slow Production Speed

Although MJT 3DP excels in producing high-resolution and complex models, its volumetric throughput tends to be slower compared to other 3D printing methods. For applications requiring rapid prototyping or on-demand production, such as surgical planning, this slower speed can hinder timely interventions and limit the technology’s effectiveness in acute care settings.

### 3.20. Integration into Clinical Workflow

Successful integration of MJT 3DP into existing clinical workflows may pose logistical challenges. This includes establishing standard operating procedures, ensuring compatibility with imaging technologies, and training staff to utilize the technology effectively. Resistance to change and the inertia of established practices can further complicate the adoption process.

## 4. Conclusions

The existing literature on MJT 3DP, combined with our practical experience at the University of Cincinnati, underscores the technology’s significant contributions to the treatment of a wide array of medical conditions. This is primarily attributed to its capacity to produce full-color models with high surface quality, facilitating enhanced visualization and understanding of complex anatomical structures. Additionally, MJT is a highly accurate 3DP technology as evidenced by the data reported in the literature which demonstrates an accuracy at par or superior to VP 3DP technology. However, MJT 3DP faces several hurdles in its utilization within medicine. Firstly, MJT 3D printers are expensive, and can be priced from $39,000 (Mimaki^®^ 3DUJ-2207) to $649,000 (XJet^®^ Carmel 1400C). The price range significantly varies based on the machine’s capabilities and features. Second, the cost of materials is higher compared with more accessible technologies such as MEX with the cost as high as 67 times [91]. Third, MJT 3D printers are typically larger; therefore, they can be more complex internally, which may require a certain degree of expertise both in operation and in maintenance. Fourth, MJT 3D printing technology, like other 3DP technologies, suffers from a slow volumetric throughput and larger models can take several days to fabricate. This could pose a problem for urgently scheduled procedures. Finally, although there have been advancements in materials, the material properties including color and tactility are still not very similar to the native tissue that are visualized and manipulated in the operating room during an actual surgery. As these technological bottlenecks are slowly addressed, the technology will likely penetrate further into healthcare and make available additional applications previously unimagined.

## Figures and Tables

**Table 1:** Medical applications of MJT 3DP in addition to the specific 3D printer used in each publication as well as material. ‘*’ Publications do not detail either material or 3D printer utilized.

Human Anatomy and Application	3D Printer Specific Technology with Materials	MJT 3DP Specific Application	Medical Device	Ref.
Liver	Objet Connex 350; TangoPlus, VeroBlue, VeroClearPlus, TangoBlackPlus	Pre-operative planning for LDLT	Anatomic Model	[29]
Liver	Objet 500 Connex 3; Acrylic Resin	Pre-operative planning for pediatric hepatoblastoma	Anatomic Model	[30]
Liver	Objet Connex500; TangoPlus, TangoBlackPlus	Pre-operative planning for LDLT in infants	Anatomic Model	[31]
Liver	Objet Eden 350V; TangoPlus, TangoBlack	Feasibility study for printing liver models	Anatomic Model	[32]
Prostate	*	Surgical planning for robotic assisted radical prostatectomy	Anatomic Model	[33]
Kidney	Objet 500 Connex 3 *	Patient education of kidney and kidney tumor anatomy	Anatomic Model	[34]
Kidney	Objet Connex3 *	Intra-operatively assistance in complex kidney stone case	Anatomic Model	[35]
Pelvis	Stratasys J750 *	Surgical planning for endometriosis		[36]
Kidney	*	Pre-operative and intra-operative assistance in robotic assisted partial nephrectomy	Anatomic Model	[37]
Prostate	*	Feasibility/utility of prostate tumor models	Anatomic Model	[38]
Oral; Cranio-maxillofacial	Objet Connex 350; Molding Silicone	Pre-operative planning for complex intracranial aneurysms	Anatomic Model	[39]
Oral; Cranio-maxillofacial	Objet 260 Dental Selection; VeroWhite, VeroMagenta, VeroBlack	Pre-operative planning of complex deformities of the skull base and craniovertebral junction	Anatomic Model	[40]
Oral; Cranio-maxillofacial	Objet 350 Connex *	Pre-operative planning of pediatric mastoid surgery	Anatomic Model	[41]
Oral; Cranio-maxillofacial	Spectrum Z 510 3D Color Printer *	Pre-operative planning of surgery treating mandibular prognathism	Anatomic Model	[42]
Oral; Cranio-maxillofacial	Objet 350 Connex 3; VeroCyan, VeroMajenta, VeroYellow	Pre-operative planning of skull base and tumor surgery	Anatomic Model	[43]
Ophthalmology	Objet *	Fitting implants pre-operatively for surgery treating orbital floor fractures	Surgical Template; Surgical Guide	[44]
Ophthalmology	Projet 660 Pro *	Intra-operative assistance for orbital defect reconstruction	Surgical Template	[45]
Ophthalmology	Projet 3510 HD *	Eye model for fundus viewing	Anatomic Model	[46]
Ophthalmology	3D Systems Z650; Visijet C4 Spectrum	Dissection eye model for medical student training	Anatomic Model	[47]
Ophthalmology	Objet Connex 350; MED 610	Eye prosthesis used in patient with acquired anophthalmos	Prosthesis	[48]
Ophthalmology	*	Ocular prosthetic in patient case	Prosthesis	[49]
Ophthalmology	Objet 30 Prime; MED610	Eye crutches for blepharoptosis	Prosthesis	[50]
Radiology	Objet Eden 500V; VeroClear	Molecular imaging phantoms including liver with liver tumor	Radiologic Phantom	[51]
Radiology	Objet 500 Connex 3; Vero Pure White, Flexible Agilus30 Clear	Anthropomorphic thorax phantom	Radiologic Phantom	[52]
Radiology	*	Thyroid cancer phantom	Radiologic Phantom	[53]
Radiology	Objet 500 Connex 3; VeroClear, TangoPlus, Vero Pure-White	Soft tissue phantoms	Radiologic Phantom	[54]
Radiology	*	Cardiovascular phantoms	Radiologic Phantom	[55]
Radiology	Tangoplus *	Phantom of glenohumeral joint	Radiologic Phantom	[56]
Radiology	MED610, TangoPlus, VeroWhite *	Best MJT filler compound to achieve radiopaqueness	----------------	[57]
Radiology	VeroClear, Tango *	Imagining properties of MJT materials	----------------	[58]
Pulmonology	Objet 500 Connex 3; FullCure RGD851, VeroMagenta, FullCure 930, TangoPlus	Bronchoscopic simulator	Surgical Simulator	[59]
Pulmonology	Objet 500 Connex 3; Vero Color, Aglius	Thoracoscopic simulator	Surgical Simulator	[60]
Neurosurgery	Stratasys J750; SUP706, Bone^TM^, Skull^TM^	Burr hole procedure simulator	Surgical Simulator	[61]
Plastic surgery	Objet 500; Shore A75, Shore A85	Rhinoplasty simulator	Surgical Simulator	[62]
Otolaryngology	Objet 500 Connex; VeroWhitePlus, TangoPlus	Endoscopic sinus surgical simulator	Surgical Simulator	[63]
Otolaryngology	Stratasys J720 Dental; VeroWhitePlus, VeroMagenta, Agilus30	Endoscopic sinus surgical simulator	Surgical Simulator	[64]
Otolaryngology	Objet Connex 500 *	Endoscopic skull base surgical simulator	Surgical Simulator	[65]
Otolaryngology	Objet 350 Connex *	Temporal bone surgical simulator	Surgical Simulator	[66]
Dental	Objet Connex 350; Acrylic Based Resin	Dental implant surgical simulator	Surgical Simulator	[67]
Dental	Objet 30 Prime; MED610	Fabrication of tooth to be used in transplantation	Implant	[68]
Otolaryngology	Objet Connex; Materialize Heartprint^TM^	Feasibility of models replicating laryngotracheal stenosis	Anatomic Model	[69]
Cardiology	Objet 500 Connex 3 *	Heart model to be used in student education	Anatomic Model	[70]
Emergency medicine	Objet 500 *	Mapping chest wall stability for thoracotomy	Anatomic Model	[71]
Dental	Stratasys J750; Agilus30	Oral sports mouth guard	Unclassified	[72]
Orthopedics	Objet 350 Connex 3 *	Pre-operative planning of surgery treating musculoskeletal tumors	Anatomic Model	[73]

**Table 2:** Listed publications that involved accuracy studies are provided for reference. Each publication includes data of the specific 3D printers involved, the materials involved, as well as accuracy results. However, 3D printer and material data were not included for systematic/meta-analysis research articles investigating accuracy. ‘*’ The listed publication did not specify materials involved in the study.

Description	Three-Dimensional Printer(s) and Materials	Accuracy Results	Reference
Surgical template accuracy between VP, SLS, and MJT using scanning of printed object and comparing with designed files	(VP—SLA) Form 2; Dental SG Resin (MJT) Objet Eden260VS; MED610 (SLS) ProX DMP 200; LaserForm Co-Cr	MJT was concluded to have the greatest accuracy and highest reproducibility	[80]
Surgical guides printed with MJT—PolyJet and multijet—and VP technology compared using four different caliper measurements compared with designed files	(VP – SLA) Form 2; Clear Resin (MJT) Objet 500 Connex3; Vero Magenta (MJT) ProJet 3510 SD; VisiJet Cristal	The guide printed with the Objet 500 Connex3 (MJT) was considered to have greatest accuracy compared with the ProJet 3510 SD (MJT) and VP 3D printer	[81]
35 models of large and small vessel were printed using MEX, and MJT. The models were than analyzed for accuracy using CT scanning and comparing model formed from resulting DICOM with original STL file.	(MEX) Ultimaker 2; Polylactic Acid (PLA) (MJT) Objet 30 Prime; Tango Series	MJT printing technology was considered to be comparable in accuracy with VP	[84]
Meta-analysis accuracy study in 2021 comparing accuracy between the various 3DP technologies	-	Revealed that MJT and SLS 3DP offered the lowest absolute mean difference in terms of accuracy (0.09 mm)	[82]
Comparison of accuracy between MJT – PolyJet and multijet and VP in mandibular surgical templates	(VP—SLA) Form 2; Dental SG Resin (MJT) Objet Eden260VS; Veroclear (MJT) ProJet 3500; VisiJet Stoneplast	Found that the 3DP technology had no significant effect on the accuracy of guided mandibular implant surgery	[85]
Comparison of accuracy between MJT, MEX, and VP 3DP technology for drill guides using scanner	(VP—SLA) Form 3; Dental SG Resin (VP—DLP) Wanhao Duplicator 7 Plus; Freeprint ortho 405 (MJT) Objet30 Prime; MED610, SUP705 (MEX) Ultimaker 3 Extended; Nylon680, ProFill^TM^ polyvinyl alcohol	MJT and VP 3DP offered the greatest accuracy for the drill guides; however, there was no significant difference in accuracy between them	[90]
Comparison of 3DP technology accuracy between MJT and VP for retainers using landmark measurements	(VP—SLA)—Form 3 * (VP—DLP)—Moonray * (VP—cDLP)—Envision One cDLM Dental * (MJT)—Objet Eden260VS *	The VP and MJT technologies were concluded to have no significant difference in accuracy for 3DP retainers	[87]
Accuracy of dental surgical guides between VP, MEX, SLS, and MJT	(VP—SLA)—Form 2 * (VP—DLP) – Rapid Shape D40 * (VP—DLP)—Cara Print 4.0 * (MJT)—Stratasys J750 * (MEX)—Raise 3D Pro2 * SLS—Prodways P1000 *	VP and MJT technologies were concluded to have no significant difference between each other	[89]
Accuracy of dental maxillary and mandibular guides between VP and MJT	(VP—SLA)—Form 2 * (VP—DLP)—Juell 3D * (VP—DLS)—Carbon M2 * (MJT)—Objet Eden 260VS *	No significant difference between guides fabricated with MJT and VP was found	[83]
Systematic search of accuracy in full-arch dental models formed from VP, MEX, and MJT	-	The accuracy between the VP and MJT 3DP technologies did not portray a significant difference	[88]

## Data Availability

Not applicable.
